# First Report on *Microcystis* as a Potential Microviridin Producer in Bulgarian Waterbodies

**DOI:** 10.3390/toxins13070448

**Published:** 2021-06-28

**Authors:** Blagoy Uzunov, Katerina Stefanova, Mariana Radkova, Jean-Pierre Descy, Georg Gärtner, Maya Stoyneva-Gärtner

**Affiliations:** 1 Department of Botany, Faculty of Biology, Sofia University, 8 blvd. Dragan Zankov, 1164 Sofia, Bulgaria; 2 AgroBioInstitute, Bulgarian Agricultural Academy, 8 blvd. Dragan Zankov, 1164 Sofia, Bulgaria; katerina_stefanova@abi.bg (K.S.); marianaradkova@yahoo.com (M.R.); 3 Unité d’Océanographie Chimique, Université de Liège, Sart Tilman, 4000 Liège, Belgium; jpdescy@uliege.be; 4 Institut für Botanik der Universität Innsbruck, Sternwartestrasse 15, 6020 Innsbruck, Austria; georg.gaertner@uibk.ac.at

**Keywords:** coastal lake, Cyanobacteria, Cyanoprokaryota, cyanotoxins, harmful algal blooms, *Microcystis aeruginosa*, reservoir

## Abstract

Bulgaria, situated on the Balkan Peninsula, is rich in small and shallow, natural and man-made non-lotic waterbodies, which are threatened by blooms of Cyanoprokaryota/Cyanobacteria. Although cyanotoxins in Bulgarian surface waters are receiving increased attention, there is no information on microviridins and their producers. This paper presents results from a phytoplankton study, conducted in August 2019 in three lakes (Durankulak, Vaya, Uzungeren) and five reservoirs (Duvanli, Mandra, Poroy, Sinyata Reka, Zhrebchevo) in which a molecular-genetic analysis (PCR based on the precursor *mdnA* gene and subsequent translation to amino acid alignments), combined with conventional light microscopy and an HPLC analysis of marker pigments, were applied for the identification of potential microviridin producers. The results provide evidence that ten strains of the genus *Microcystis*, and of its most widespread species *M. aeruginosa* in particular, are potentially toxigenic in respect to microviridins. The *mdnA* sequences were obtained from all studied waterbodies and their translation to amino-acid alignments revealed the presence of five microviridin variants (types B/C, Izancya, CBJ55500.1 (*Microcystis* 199), and MC19, as well as a variant, which was very close to type A). This study adds to the general understanding of the microviridin occurrence, producers, and sequence diversity.

## 1. Introduction

Currently, the problems caused by harmful algal blooms (HABs) of Cyanoprokaryota/Cyanobacteria (hereafter abbreviated as CyanoHABs) and their toxins (cyanotoxins) are recognized worldwide [1,2,3]. However, since the beginning of the 21st century, a series of studies have demonstrated that the assessment of cyanobacterial toxicity cannot solely rely on the commonly known cyanotoxins, implying the activity of other compounds, the structure and function of which need elucidation (for details see [4]). In this regard, beyond the best studied and routinely monitored cyanotoxins from the specific group of cyclic non-ribosomally produced heptapeptides, named microcystins [1,2,3], more than six hundred other peptides or peptidic metabolites, commonly unified as cyanopeptins (CNPs), were isolated from the vast metabolite repertory of cyanoprokaryotes [5,6,7,8]. Despite some variations in composition and amounts, these CNPs were regularly found during CyanoHABs [7]. Nowadays, CNPs are gaining strong attention because it has been reported that metabolomic profiles consisting of different CNPs may affect the invertebrates and fish populations [7]. The CNPs include the unique 16-membered family of the toxins microviridins (MVs), named after their first discovered producer—*Microcystis viridis* (A. Braun) Lemmermann (strain NIES-102) [9]. MVs are peculiar ribosomally synthesized and post-translationally modified peptides (depsipeptides), most of which inhibit serine proteases and have been found in different species of aquatic *Microcystis* Lemmermann, *Anabaena* Bory ex Bornet et Flahault s.l., *Planktothrix* Anagnostidis et Komárek (Syn. *Oscillatoria* Vaucher ex Gomont p.p.), *Nodularia* Mertens ex Bornet et Flahault and *Radiocystis* Skuja, and in some aeroterrestrial/freshwater strains of *Nostoc* Vaucher ex Bornet et Flahault (e.g., [8,9,10,11,12,13,14,15,16,17,18,19,20,21,22,23,24,25,26,27,28,29,30,31,32,33,34,35,36,37,38]). Some features given to MVs were suggested to be related to their allelochemical properties and to the affecting of proteolysis, in particular [8,39]. However, further research in this field is needed to clarify the function and potential ecological role of MVs (e.g., [8,35]). The biosynthesis of MVs was elucidated and it was demonstrated that the microviridin (MV) biosynthetic clusters have different organizations [8,18]. Although the subsequent application of the PCR approach revealed a far more widespread occurrence of MV genes than expected, and their global distribution was suggested, the natural diversity of MV precursor sequences remained almost unknown and MVs have been detected infrequently in cyanoprokaryote peptide screenings [8,18].

Bulgaria, situated on the Balkan Peninsula, has numerous (ca. 10,000) wetlands; however, they occupy less than 1% of its territory and, being mostly shallow, small, and located in lowlands or plains, are vulnerable to threats of CyanoHABs [40]. Although the first records on algal flora dated from the end of the 18th century and targeted investigations on cyanotoxins started after 2000 [41], there are no studies on MVs or their producers in the country. This paper provides first data on the presence and diversity of potential MV producers, and on their contribution to the phytoplankton in eight selected Bulgarian waterbodies, obtained after combined studies by conventional light microscopy (LM), HPLC pigment marker analysis, and molecular-genetic studies. The latter include PCR amplification of the entire MV precursor gene A (*mdnA*) [8,18], the translation of obtained nucleotide sequences to amino acids, and the determination of their alignment with the intention to identify the MV types. The results provide evidence for ten different *Microcystis* strains as potential producers of five MV variants.

## 2. Results

### 2.1. Phytoplankton Species Composition and Abundance, Obtained by Light Microscopy (LM)

**Figure 1:**
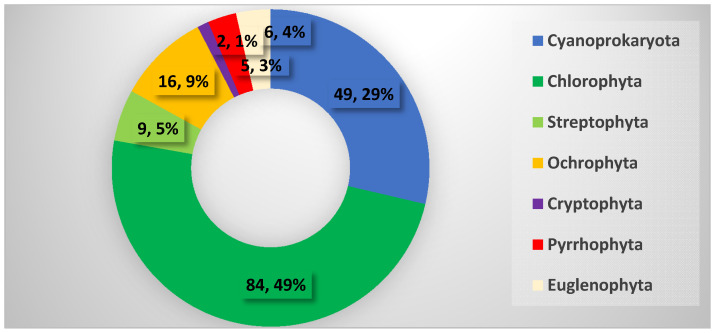
The general phytoplankton species composition in eight waterbodies in Central and Eastern Bulgaria sampled in August 2019. The number of identified taxa and their percentage contribution to the total phytoplankton biodiversity in each phylum is indicated.

In total, 171 species were identified using LM in the phytoplankton of the eight studied waterbodies. They belonged to seven algal phyla, namely Cyanoprokaryota, Chlorophyta, Streptophyta, Ochrophyta, Cryptophyta, Pyrrhophyta, and Euglenophyta (Figure 1). Cyanoprokaryotes, represented by 49 species, had an important contribution to the total phytoplankton biodiversity (29%), occupying second position after Chlorophyta (49%).

**Figure 2:**
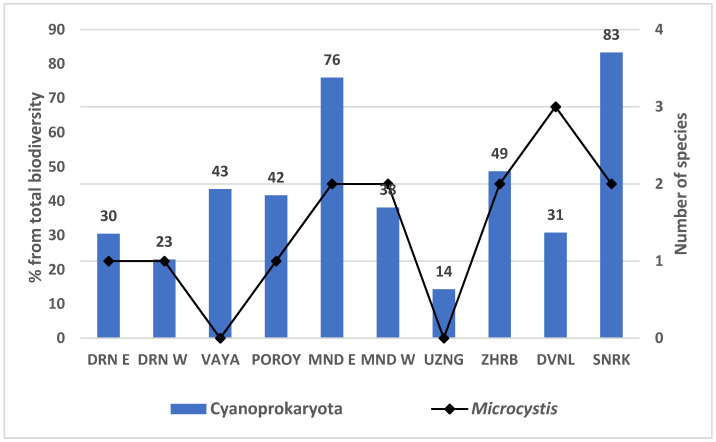
The relative contribution of Cyanoprokaryota to the phytoplankton biodiversity (total number of species) by sites in the studied Bulgarian non-lotic waterbodies (right axis) and number of certainly identified by light microscopy morphospecies of *Microcystis* (left axis). Abbreviations: DRN E—Lake Durankulak (site East), DRN W—Lake Durankulak (site West), MND E—reservoir Mandra (site East), MND W—reservoir Mandra (site West), UZNG—Lake Uzungeren, ZHRB—reservoir Zhrebchevo, DVNL—reservoir Duvanli, SNRK—reservoir Sinyata Reka.

The total number of phytoplankton species ranged between six (reservoir Sinyata Reka) and 65 (reservoir Duvanli), and the number of cyanoprokaryotes was between five (reservoir Poroy) and 25 (reservoir Mandra, site East). They contributed from 14% (lake Uzungeren) to 83% (reservoir Sinyata Reka) of the total biodiversity at each site (Figure 2).

**Figure 3:**
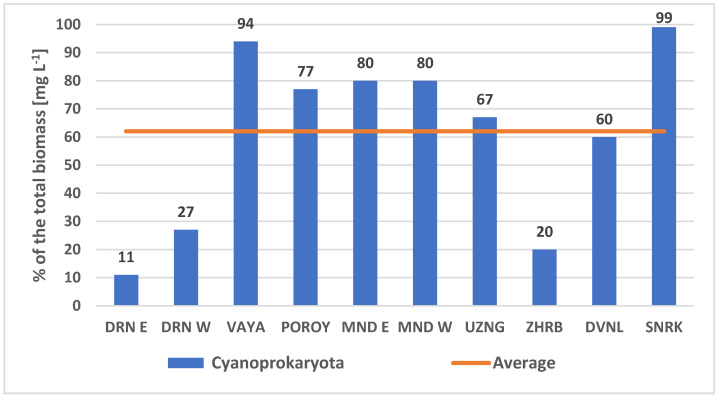
The relative contribution of Cyanoprokaryota to the total phytoplankton biomass, estimated from microscope counts, in the phytoplankton of the studied Bulgarian non-lotic waterbodies (August 2019). Legend abbreviations: DRN E—Lake Durankulak (site East), DRN W—Lake Durankulak (site West), MND E—reservoir Mandra (site East), MND W—reservoir Mandra (site West), UZNG—Lake Uzungeren, ZHRB—reservoir Zhrebchevo, DVNL—reservoir Duvanli, SNRK—reservoir Sinyata Reka.

Cyanoprokaryotes comprised from 11% (Lake Durankulak, Eastern site) to 99% (reservoir Sinyata Reka) of the total phytoplankton biomass (Figure 3). On average they contributed 62% to the total phytoplankton biomass (Figure 3). In most sites where the cyanoprokaryote biomass exceeded this average value, the phytoplankton was dominated by filamentous species: *Aphanizomenon* cf. *klebahnii* Elenkin ex Pechar (dominant in all sites of the coastal reservoirs Mandra and Poroy), *Sphaerospermopsis torques-reginae* (Komárek) Werner, Laughinghouse IV, Fior et Sant’Anna (dominant in the inland reservoir Sinyata Reka), *Planktothrix isothrix* (Skuja) Komárek et Komárková and *P. suspensa* (Pringsheim) Anagnostidis & Komárek (co-dominants in the coastal Lake Vaya), *Pseudanabaena limnetica* (Lemmermann) Komárek and *Spirulina* cf. *laxissima* G. S. West (co-dominants in the inland reservoir Duvanli).

Considering the results on potential MV-producers, obtained by the PCR analysis, below we present in more detail the LM data on the genus *Microcystis,* from which four species (morphospecies) were identified: * M. aeruginosa* (Kützing) Kützing, *M.* cf. *comperei* Komárek, *M. natans* Lemmermann ex Skuja, and *M. wesenbergii* (Komárek) Komárek (Table 1). In some samples we also found separate cells, disintegrated colonies, or initial colonies, as well as colonies with transitional morphology, for which species identification was not possible (Table 1). *Microcystis* was observed in almost all waterbodies (except Vaya and Uzungeren) with a low number of certainly identified species, ranging in separate sites between one (in the reservoir Poroy) and three (in the reservoir Duvanli) (Figure 2, Table 1). Its contribution to the total phytoplankton biomass was different, but always extremely low—between <0.05% and <1% (Table 1). The only exception was the higher amount of *M. aeruginosa* (<5%) in the eastern part of the reservoir Mandra (Table 1).

### 2.2. Results on General Phytoplankton Composition from HPLC Analysis of Marker Pigments

**Figure 4:**
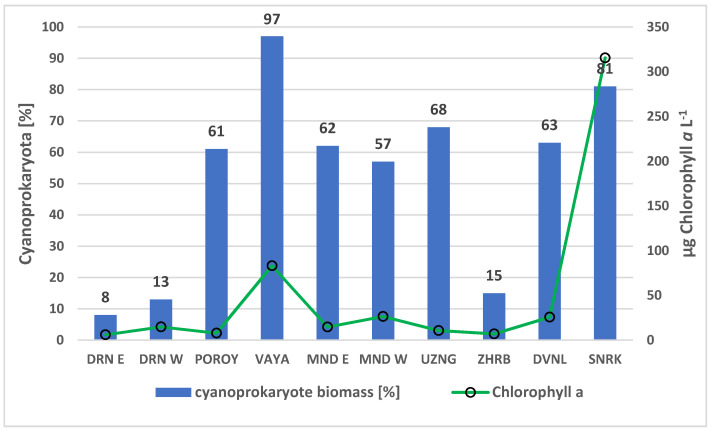
The cyanoprokaryote contribution to the total phytoplankton biomass according to the pigment markers, analyzed by HPLC (expressed as percentage contribution to chlorophyll *a*, calculated using CHEMTAX) and chlorophyll *a* concentration in the studied non-lotic Bulgarian waterbodies (August 2019). Abbreviations: DRN E—Lake Durankulak (site East), DRN W—Lake Durankulak (site West), MND E—reservoir Mandra (site East), MND W—reservoir Mandra (site West), UZNG—Lake Uzungeren, ZHRB—reservoir Zhrebchevo, DVNL—reservoir Duvanli, SNRK—reservoir Sinyata Reka.

According to HPLC determination of marker pigments concentrations, processed with CHEMTAX for estimating phytoplankton class abundance [42,43,44,45], cyanoprokaryotes dominated the phytoplankton of most waterbodies (Figure 4**)**.

The values of chlorophyll *a* indicated the meso- to hypertrophic status of the studied waterbodies. The chlorophyll *a* concentration ranged between 6 (coastal lake Durankulak, site East) to 316 µg L^−1^ (reservoir Sinyata Reka) and 83 µg L^−1^ (coastal lake Vaya). The contribution of cyanoprokaryotes to the phytoplankton biomass ranged in a similar way, being the lowest in the eastern part of Durankulak (8%) and the highest in Vaya (97%) and Sinyata Reka (81%) (Figure 4).

### 2.3. Results from PCR Analysis for Microcystin-Producing Strains

**Figure 5:**
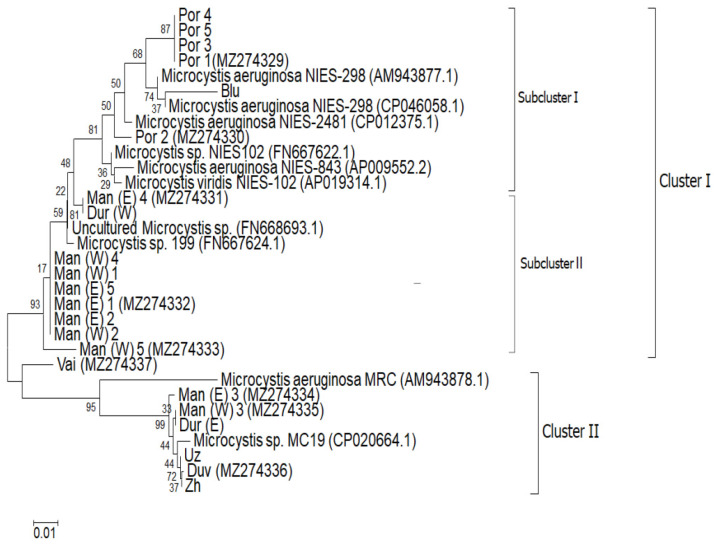
The neighbor-joining phylogenic tree constructed with sequences, obtained from the three libraries from the coastal reservoirs Mandra (sites East and West) and Poroy, combined with the sequences from the PCR fragments, amplified from all studied Bulgarian waterbodies. The bootstrap value is a percentage of 1000 resamplings. The new obtained sequences are supplied with the NCBI accession numbers MZ274329-MZ274337. For the identical sequences (IS), obtained during this study, only one accession number is provided in each cluster or subcluster as follows: (1) The IS from Poroy (Por 1, 3-5)—MZ274329; (2) The IS from Durankulak (Dur W) and Mandra (Man (E) 4)—MZ274331; (3) The IS from Mandra (Man (W) 1,2,4 and Man (E) 1,2,5)—MZ274332; (4) The IS from Duvanlii (Duv) and identical sequences from Uzungeren (Uz) and Zhrebchevo (Zh)—MZ274336. The partial sequence Blu (131 bp) from the reservoir Sinyata Reka (=Blue River) has no NCBI number, but is generally similar to Por1 (explanations are provided in the text).

The precursor gene *mdnA* from the MV synthetized gene cluster [18] was successfully amplified in all ten investigated metagenomic DNA samples from the studied waterbodies and 22 sequences were obtained. Checking these in the National Centre for Biotechnology Information (NCBI) genetic database [46] by the standard Basic Local Alignment Search Tool (BLAST) [47], revealed ten different sequences, which represent ten strains. They showed high homology (97–100%) with different *mdnA*-containing *Microcystis* strains, published in NCBI [46]. All obtained sequences with their corresponding highly homologous NCBI strains are divided in two clusters in the constructed phylogenetic tree (Figure 5).

The first cluster contains two clearly defined subclusters. Subcluster I is formed mainly by the sequences, isolated from the reservoirs Poroy and Sinyata Reka, which show high homology (98.7–99.1%) with two strains of *M. aeruginosa*—NIES-298 and NIES-2481. Subcluster II contains most of the sequences (eight), isolated from the reservoir Mandra, which show 97.3–100% homology with two *Microcystis* strains unidentified to species level (*Microcystis* sp. FN 668693.1 and *Microcystis* sp. 199), the first of which was uncultured.

The second cluster contains sequences, which show high homology (99.1–99.6%) with the strain *Microcystis* sp. MC19. They are isolated from five different waterbodies, including two sequences from both sides of the reservoir Mandra, and single sequences from the reservoirs Duvanli and Zhrebchevo, and from the coastal lakes Durankulak and Uzungeren as well.

Interestingly, the isolate from the coastal Lake Vaya differs from all other obtained sequences and the two single sequences obtained from both sites of Lake Durankulak are quite different: the sequence from the western part of the lake is situated in cluster I, whereas the sequence from the eastern part of the same lake is positioned in cluster II (Figure 5).

We intended to identify the MV variants through the translation of the obtained *mdnA* sequences and their comparison with published leader peptide sequences and amino acid alignments [8,18], processed by the Vector NTI Advance 11.5 software package (Version 11.5, Invitrogen Corporation, Carisbad, CA 92008, USA, 2010) and with part of the consensus sequence designed by WebLogo [48].

**Figure 6:**
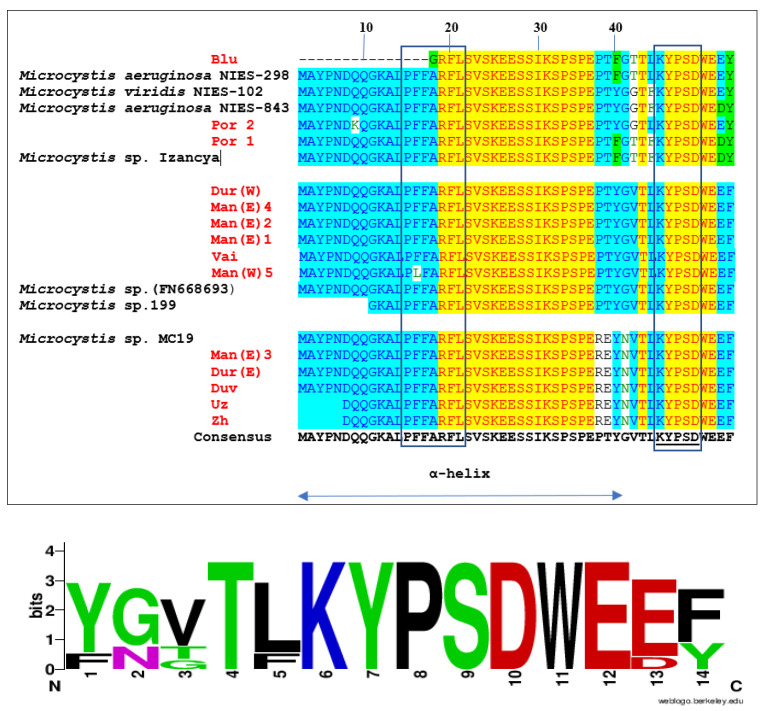
Comparison of the amino acid alignment of the translated *mdnA* sequences, isolated from eight Bulgarian waterbodies (red font), with the alignments of the translated *mdnA* sequences in NCBI strains of *M. aeruginosa* and *M. viridis* (black font), processed by the Vector NTI v.10 software package. Below is a part of the consensus sequence generated by WebLogo [48]. The specific motif PFFARFL from the α-helix of the leader peptide and the core motif KYPSD are outlined. Legend: Blu—reservoir Sinyata Reka (=Blue River); Dur (E)—Lake Durankulak, site East; Dur (W)—Lake Durankulak, site West; Duv—reservoir Duvanli; ManE—reservoir Mandra, site East; Man (W)—Mandra West part; Por—reservoir Poroy; Vai—Lake Vaya; Uz—Lake Uzungeren; Zh—reservoir Zhrebchevo.

The determined MV sequences (Figure 6) show that most of our strains should be capable of producing MV with the highly conserved PFFARFL motif from the α-helix of the MV leader peptide structure [8] (Figure 6). The exceptions are in: (1) the partially sequenced strain Blu from the small inland reservoir Sinyata Reka, which contained less amino acids in comparison with all other strains and in which only GRFL was detected in the α-helix; (2) the strain Man(W) 5, isolated from the Mandra reservoir, in which PLFARFL was found in the α-helix (Figure 6). The KYPSD sequence from the standard MV consensus sequence TXK(Y/W/F)PS DW(E/G)(E/D), firstly named as the MV core motif [18], and later represented mostly as the larger canonical TxKxPSD motif [8,25], was found in all *mdnA* sequences isolated in this study (Figure 6). The order of the neighboring amino acids before and after this core motif differed in the obtained strains (Figure 6) and was compared with the standard published parts of MV sequences [8,18] (Table 2).

The comparison of data demonstrated that:

(1) The peculiar MV sequence with the shorter α-helix, obtained from the strain Blu, isolated from the small inland reservoir Sinyata Reka, was close but not identical with the MV sequence from *M. aeruginosa* NIES-298 (Figure 6). According to the alignment of amino acids neighboring to KYPSD, both sequences (Blu and NIES298) coincided completely with MV variant B/C (Table 2);

(2) The MV sequence from the single isolate Por 2 from the small coastal reservoir Poroy, was very close but not identical to the MV variants of *M. viridis* NIES 102 and *M. aeruginosa* 843 (Figure 6). According to the sequences neighboring to KYPSD, it was very close to MV variant A (Table 2);

(3) The MV sequence of the strain Por 1 (and of its identical strains Por 3,4,5) from the same Poroy reservoir completely coincided with the MV variant, isolated from *Microcystis* sp. strain *Izancya* (FN667620.1) and named MV Izancya [18] (Figure 6; Table 2). This MV has not been referred to as one of the main MV variants, published by do Amaral et al. [8];

(4) Most MV sequences obtained from Mandra, as well as the sequences from Vaya and Durankulak (western part) are identical with the alignment of *Microcystis* sp. FN668693 (except the above-mentioned Man (W) 5). They all are very close to the MV of *Microcystis* sp. 199, which, due to a partially known sequence [46] has a shorter published α-helix (Figure 6). The MV of *Microcystis* sp. 199, named MV 199 [18], later has been referred to as a separate MV variant, labelled as CBJ55500.1 (*Microcystis* 199) [8]. According to the alignment of amino acids neighboring to KYPSD, all MV sequences of the strains Dur (W), Man (E) 1,2,4, Man (W) 5, and Vai coincide completely with the MV variant CBJ55500.1 (*Microcystis* 199) (Table 2).

(5) The five MV sequences, obtained from the strains isolated from Durankulak (eastern part), Duvanli, Mandra (eastern part), Uzungeren and Zhrebchevo, are similar to the MV variant of the strain *Microcystis* sp. MC19, despite the last two having a shorter α-helix (Figure 6). According to the alignment of amino acids neighboring to KYPSD, all MV sequences isolated from the strains Man (E) 3, Dur (E), Duv, Uz, and Zh are completely similar with one of the MV variants, identified from field samples by Ziemert et al. [18] (Table 2).

## 3. Discussion

The results obtained during this study provide the first evidence for the presence of potential MV producers in Bulgarian waterbodies. According to the distribution of the precursor gene *mdnA*, all ten new potential MV-producing strains belonged to the genus *Microcystis* and to its species *M. aeruginosa* in particular (Figure 5). This morphospecies was the most frequent in the studied waterbodies, especially when considering that some of the separate cells and disintegrated or transitional colonies may also belong to it (Figure 1, Table 1). This consideration can explain the PCR result for the *mdnA* sequence in all waterbodies, including those for which certain *Microcystis* morphospecies have not been indicated (Figure 5). Our finding of *M. aeruginosa* as the most common potential MV producer is in complete agreement with the conclusion in the summary of Amaral et al. [8] (p.14), who stated that “the genus *Microcystis* and the species *M. aeruginosa* are the largest producers of microviridins—currently, of the 25 isolated microviridins, 11 belong to the genus *Microcystis*, and eight of these belong to the species *M. aeruginosa*”. At present, it is impossible to refer to the three other *Microcystis* species detected by LM (*Microcystis* cf. *comperei*, *Microcystis natans*, and *M. wesenbergii*) as potential MV producers. However, it should be noted that some of the obtained sequences belong to three unidentified *Microcystis* strains, available in NCBI [46] (Figure 5). Interestingly, the single strain Por 2, isolated from the small coastal reservoir Poroy and its MV sequence are close, but not identical to the first known MV producer and its MV—*M. viridis* NIES-102. By LM *M. viridis* was not identified during this study nor in our samples from the same coastal wetlands Durankulak, Poroy, Uzungeren, Mandra and Vaya, as well as in the inland reservoir Sinyata Reka, collecte d in the previous year, 2018 [49]. This species was, however, present but rare in the samples from the coastal Lake Vaya collected in August 2004 [50,51].

We did not obtain any PCR signal in relation with the *mdnA* gene and other cyanoprokaryote species, found as dominants during the blooms (Figure 3), namely *Aphanizomenon* cf. *klebahnii*, *Sphaerospermopsis torques-reginae*, *Planktothrix isothrix* and *P. suspensa*, *Pseudanabaena limnetica*, and *Spirulina* cf. *laxissima.* This result agrees with the fact that these algae have not been pointed out as potential MV producers [8,9,11,12,13,15,16,19,23,26,27,28]. However, Ziemert et al. [18] (p. 3570) stressed that “The fact that no PCR product was obtained thus does not necessarily imply that the strains lack the capacity to produce microviridin-like peptides. Rather, it is possible that the orthologous genes in these strains are in a different order or in an independent position in the genome”. Therefore, future genetic and biochemical studies are needed to identify all possible MV producers.

The translation, applied to the *mdnA* sequences, revealed four variants of MVs, which could be related with certainty to known MV types: B/C, MV from *M. isancya*, CBJ55500.1 (*Microcystis* 199), and MV from *Microcystis* MC19. One more MV variant was very close, but not identical to MV type A and to MVs, isolated from *M. viridis* NIES 102 and *M. aeruginosa* 843 [8,18] (Figure 6, Table 2).

All results on the geographic diversity of *Microcystis* strains, obtained both by LM and molecular-genetic methods, as well as on the potential release of five MV variants, occurred in a context of high cyanoprokaryote contribution to phytoplankton biodiversity and biomass, as shown by LM and HPLC analyses (Figure 2, Figure 3 and Figure 4). All observations point to the meso- to hypertrophic character of the eight studied waterbodies and once more stress the strong vulnerability to CyanoHABs of the shallow lowland and plain wetlands of Bulgaria [40,41], in which four general types of cyanotoxins (microcystins, anatoxins, saxitoxins, and cylindrospermopsins) have been found (e.g., [41,52]). Although *Microcystis* was found in small amounts, likely due to strong rains preceding the sampling [53], this genus was commonly reported among the most widespread causative agents of CyanoHABs in Bulgaria [41,51] and its mass development in dry summer periods is to be expected. Therefore, the finding of its MV-producing strains in the country stresses the need for future larger scale studies of these peculiar cyanotoxins.

## 4. Materials and Methods

### 4.1. Sites and Sampling

**Figure 7:**
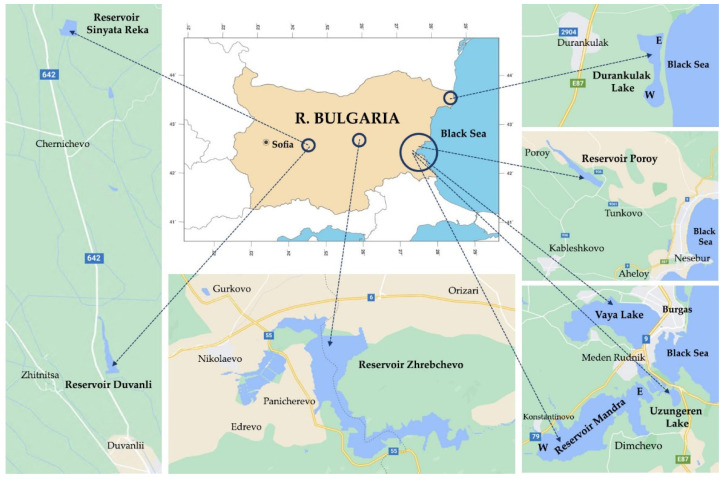
Map of R. Bulgaria with locations of the studied waterbodies (in case of different sampling sites, they are labelled as E and W) (modified after Ginkgo Maps (http://www.ginkgomaps.com (assessed on 26 May 2021)) and Google Maps (https://www.google.com/maps (assessed on 26 May 2021)).

The sampling was carried out in the period 14–21 August 2019 in eight waterbodies situated in Central and Eastern Bulgaria (Figure 7, Table 3). Detailed data on the morphometry, historical development, usage and conservation value of the studied three coastal lakes (Durankulak, Vaya and Uzungeren) and five reservoirs (two of which are coastal—Poroy and Mandra, and three are inland—Duvanli, Sinyata Reka, and Zhrebchevo) can be found in the Inventory of Bulgarian wetlands [40]. Here we only note that: (1) coastal lake Durankulak is a protected area and is included in the Red List of Bulgarian Wetlands [40] in the category Critically Endangered; (2) the reservoir Zhrebchevo is included in the Appendix 1 “List of complex and significant reservoirs” of the Water Act (State Gazette 67/1999) [40]; (3) the reservoirs Mandra and Zhrebchevo are large (3366 and 1851 ha, respectively), while the reservoirs Duvanli, Poroy and Sinyata Reka are small (27, 223 and 6 ha, respectively); (4) The reservoirs Zhrebchevo, Duvanli and Sinyata Reka are plain (200–600 m a.s.l.) waterbodies, while Durankulak, Poroy, Vaya, Mandra and Uzungeren are lowland lakes and reservoirs (<0–200 m a.s.l.) [40]—Table 3.

These water bodies were chosen according to the results obtained from a larger study of cyanotoxins and their producers in 28 Bulgarian waterbodies in relation to human health risks and national security problems [53,54]. In the context of this study, sampling sites were chosen after observation of each waterbody by a drone DJI Mavic 2 Enterprise Dual Pro (DJI Technology Co., Ltd. Shenzhen, China), supplied by a photo camera and capable of measuring the surface water temperature. Therefore, from two of the selected waterbodies we sampled at two sites (Table 3). The phytoplankton sampling was performed from inflatable boats. Data on geographical coordinates, altitude, water temperature, pH, total dissolved solids, oxygen concentration, and conductivity were taken by the water monitoring instruments Aquameter AM-200 and Aquaprobe AP-2000 (Aquaread Ltd., Broadstairs, UK). Water transparency was measured with a Secchi disk. The Aqualytic AL410 Photometer from AQUALYTIC^®^ (Dortmund, Germany) was used for the ex situ measurement of total nitrogen (TN) and total phosphorus (TP) [53,54]. All obtained data are shown in Table 3.

The phytoplankton samples were collected in equal amounts of 0.5 L from the surface water layer (0–20 cm) for all types of further studies. For phytoplankton identification and counts they were fixed immediately to a 2% final formalin concentration and transported to the lab, where they were further concentrated by sedimentation to a volume of 50 mL [52,53,54]. The water samples for pigment analyses and PCR studies, within a few hours after collection, were filtered under a mild vacuum through Macherey-Nagel GF5 filters (MACHEREY-NAGEL GmbH & Co. KG, Düren, Germany) with porosity 0.4 µm and Whatman 0.45 µm cellulose filters Whatman NC45 ST/Sterile EO (Merck KGaA, Darmstadt, Germany), respectively. The obtained filters were immediately placed in 15 mL sterile plastic tubes (Falcon) and preserved in a dry ice for transportation to the labs and further treatment [53,54].

### 4.2. Phytoplankton Species Composition and Abundance Assessment by Conventional Light Microscopy (LM)

The phytoplankton species composition was identified following standard taxonomic sources, with updates from Algaebase [55] on non-permanent slides under magnification 100× and immersion using a Motic B1 microscope, supplied by a Moticam 2.0 mp camera with the Motic Images 2 Plus software program (Version 2.0, Moticam, Hong Kong, China, 2017). The subsequent algal counts were done on the same microscope using the Thoma blood-counting chamber, conducting eight counts for each site. The abundance of each species was estimated in both cell numbers and biomass, the last by using the stereometrical approximations and subsequent weight recalculation [53,54,56,57]. Since it was supposed that for the estimation of toxin concentrations the biomass is a better parameter than cell numbers [58], only biomass data were presented here.

### 4.3. Phytoplankton Composition Assessment by HPLC Marker Pigment Analysis

A pigment analysis of phytoplankton in the water samples was done by HPLC with subsequent application of CHEMTAX [43,44,45] following the standard operational procedure (SOP5), described by Descy [42] in relation to the basic guidelines for detection and monitoring of toxic cyanoprokaryotes. Pigment extraction was carried in 90% HPLC-grade acetone and all processing steps were identical to those of our previous studies in the region [52,53,54].

The use of CHEMTAX allowed estimating the contribution to chlorophyll *a* of the main algal phyla: Cyanoprokaryota (identified with two pigment types), Chlorophyta and Streptophyta (presented commonly as green algae), Ochrophyta (identified mainly as classes Chrysophyceae and Bacillariophyceae), Cryptophyta, Pyrrhophyta, and Euglenophyta.

### 4.4. Molecular-Genetic Studies

The metagenomic DNA was isolated from the filters obtained after sampling in the field. Amplification was conducted with a specific pair of primers mult fw (TCACTCGAAATTACCAGAGGAA) and mdn mult rv (GGTGTAATCAAGAAAAGTGCT), designed to specifically amplify the conserved flanking regions of the precursor gene *mdnA* from the MV cluster [18]. This gene was chosen because it has been proven that it occurs in the majority of gene clusters responsible for the biosynthesis of MVs [8]. This precursor gene is needed for the production of prepeptide (immature peptide, leader peptide), required as a first step in the production of a completely tricyclic N-acetylated MV [8,59].

For the amplification procedure, the steps described in the protocol of the manufacturer of Taq polymerase (My Taq HS mix, BIOLINE, USA Inc., Taunton, MA, USA) were followed. Firstly, a 95 °C denaturation step was applied in the duration of 3 min. It was followed by 35 cycles of denaturation at 95 °C for 15 s, annealing at 59 °C for 30 s, and synthesis at 72 °C for 25 s. The final synthesis was at 72 °C in a duration of 3 min. The PCR mix contained 10µL MyTaqHS Mix (BIOLINE), and 40 nM of each primer. The amplification reaction ran on a thermal cycler (QB-96 apparatus, Quanta Biotech, Byfleet, Surrey, UK).

The amplified fragments were purified from the gel and sent for sequencing by Macrogene Inc. (Seoul, Korea). The obtained results were compared with sequences available in the National Centre for Biotechnology Information (NCBI) genetic database [46] using a search by the standard Basic Local Alignment Search Tool (BLAST) [47]. Then, only the sequences with the highest homology were used for the construction of the phylogenetic tree by application of the program Mega 6.0 and the neighbor-joining method, with 1000 bootstrap replications [60].

Additionally, three *mdnA* libraries were developed after the cloning of fragments, isolated from the coastal reservoirs Mandra (East and West) and Poroy. Five individual clones from each library were sent for sequencing by Macrogene Inc. (Seoul, Korea) and included in the constructed phylogenetic tree. There, the accession numbers, obtained from NCBI [46] for the new *mdnA*-based strains (MZ274329-MZ274337) are shown in brackets. The partial short sequence Blu (131 bp) has no NCBI number. It is generally similar to Por1, but has three different nucleotides, which occupy the 75 (G), 81 (T), and 175 (G) positions.

## Figures and Tables

**Table 1:** Distribution of *Microcystis* taxa identified by LM and their contribution to the total phytoplankton biomass in the studied Bulgarian waterbodies (WBs) in August 2019: Res.—reservoir; MA—*Microcystis aeruginosa*; MC—*Microcystis* cf. *comperei*; MN—*Microcystis natans*; MW—*Microcystis wesenbergii*; SS/DC—separate cells or disintegrated colonies; TTs—colonies with transitional morphology; n.d.—not detected.

Waterbody	MA	MC	MN	MW	SS/DC	TTs
Lake Durankulak East	<0.5%	n.d.	n.d.	n.d.	<0.05%.	n.d.
Lake Durankulak West	n.d.	n.d.	n.d.	<1%	<0.05%.	<0.05%
Lake Vaya	n.d.	n.d	n.d	n.d	<0.05%	n.d.
Res. Poroy	n.d.	n.d.	n.d.	<0.5%.	n.d.	n.d.
Res. Mandra East	<5%	n.d.	n.d.	<0.5%	n.d.	<0.05%
Res. Mandra West	<0.1%	n.d.	n.d.	<0.5%	<0.05%.	<0.05%
Lake Uzungeren	n.d.	n.d	n.d	n.d	<0.05%	n.d.
Res. Zhrebchevo	<0.5%	n.d.	<0.05%.	n.d.	<0.05%.	n.d.
Res. Duvanli	n.d.	<0.01%	<0.1%	<1%	<0.1%	<0.05%
Res. Sinyata Reka	<0.5%	n.d.	n.d.	<1%	<0.05%.	<0.05%

**Table 2:** Comparison of standard published microviridin sequences [8,18] with the microviridin sequences, obtained from eight lowland Bulgarian waterbodies (represented in red font) according to the alignment of the amino acids before (Z1–Z3) and after (EE(D)Z4) the TX KYPSD W motif [8,18]. Those marked with an asterisk (*) indicate the difference in letters, published by different authors (Y and D in [8], and F and E in [18]). The dark yellow color marks the core motif sequence KYPSD, by yellow color are shown the not changing parts of the sequences. Identical sequences are colored in dark green, and similar but not completely identical sequences are shown in light green. The grey color marks differences in the similar sequences. Legend: Blu—reservoir Sinyata Reka (=Blue River); Dur (E)—Lake Durankulak, site East; Dur (W)—Lake Durankulak, site West; Duv—reservoir Duvanli; ManE—reservoir Mandra, site East; Man (W)—Mandra West part; Por—reservoir Poroy; Vai—Lake Vaya; Uz—Lake Uzungeren; Zh—reservoir Zhrebchevo.

Microviridin Variants/Amino Acids	Z1	Z2	Z3	T	X	KYPSD	W	E	E/D	Z4
Microviridin A	Y	G	G	T	F	KYPSD	W	E	E	Y
Por 2	Y	G	G	T	L	KYPSD	W	E	E	Y
MV *Microcystis aeruginosa* NIES 843	Y	G	G	T	F	KYPSD	W	E	D	Y
MV *Microcystis viridis* NIES 102	Y	G	G	T	F	KYPSD	W	E	E	Y
Microviridin B/C	F	G	T	T	L	KYPSD	W	E	E	Y
Blu (and *M. aeruginosa* NIES 298)	F	G	T	T	L	KYPSD	W	E	E	Y
Microviridin D/K	Y*(F)	G	N	T	M	KYPSD	W	E	D*(E)	Y
Microviridin E/F		F	S	T	Y	KYPSD	W	E	D	F
Microviridin G/H	Y	P	Q	T	L	KYPSD	W	E	E	Y
Microviridin I	Y	P	T	T	L	KYPSD	W	E	D	Y
Microviridin J		I	S	T	R	KYPSD	W	E	E	W
Microviridin L	Y	G	G	T	F	KYPSD	W	E	D	Y
Microviridin SD1684, SD1634, SD1652		T	A	T	R	KYPSD	W	E	D	Y
Microviridin LH1667		Y	S	T	F	KYPSD	W	E	D	Y
Microviridin 1777	Y	N	V	T	F	KYPSD	W	E	D	Y
CBJ55500.1 (*Microcystis* 199)	Y	G	V	T	L	KYPSD	W	E	E	F
Dur (W), Man (E) 1, 2, 4, Man (W) 5, Vai	Y	G	V	T	L	KYPSD	W	E	E	F
MV from a field sample MV/MC19	Y	N	V	T	L	KYPSD	W	E	E	F
Dur (E), Man (E) 3, Uz, Zh	Y	N	V	T	L	KYPSD	W	E	E	F
MV Izancya	F	G	T	T	F	KYPSD	W	E	D	Y
Por 1	F	G	T	T	F	KYPSD	W	E	D	Y
MV *Microcystis* NIES 100	F	G	T	T	F	KYPSD	W	E	D	F
MV *Microcystis* PCC 9805		T	S	T	R	KYPSD	W	E	E	F
MV *Microcystis* NIES 103	Y	G	G	T	F	KYPSD	W	E	E	Y
MV *Microcystis* PCC 7005	G	R	G	T	L	KYPSD	W	E	E	S
MV from a field sample		Y	S	T	R	KYPSD	W	E	E	F
MV from a field sample	A	N	V	T	L	KYPSD	W	G	E	F
MV from a field sample	Y	G	G	T	L	KYPSD	W	E	D	Y
MV from a field sample	Y	G	S	T	F	KYPSD	W	E	D	F
MV from a field sample	Y	E	V	T	L	KYPSD	W	E	E	F

**Table 3:** Sampling sites (organized by order of sampling, 14–20 August 2019) and their main environmental parameters. Legend: IBWXXXX—number of the waterbody in the Inventory of Bulgarian non-lotic wetlands [40]); Alt—altitude (m a.s.l.); Area—in ha; WT—water temperature (°C); SD—Secchi depth (m); EC—electric conductivity (S m^−1^); TD—total dissolved solids (µg L^−1^); DO—oxygen concentration (mg L^−1^); TP—total phosphorus (mg L^−1^); TN—total nitrogen (mg L^−1^). For details see the text of the paper.

Waterbody	Alt	Latitude	Longitude	Area	WT	pH	SD	EC	TD	DO	TP	TN
Lake Durankulak (IBW0216) West	2	43°40.0006′	29°32.6166′		26.5	8.89	0.6	0.974	631	7.86	0.30	0.66
East	4	43°40.5355′	28°33.0806′	369	26.7	8.91	0.6	0.105	680	6.04	0.33	0.63
Res. Poroy (IBW3038)	43	42°43.3403′	27°37.5255′	223	27.5	8.05	0.4	0.644	416	7.6	0.10	0.31
Res. Mandra (IBW1720) West	7	42°24.0295′	27°19.1194′		25.88	7.9	0.45	0.676	436	7.93	0.66	0.46
East	8	42°25.9303′	27°26.7652′	3366	27.2	8.46	0.45	0.578	375	7.87	1.5	1.8
Lake Vaya (IBW0191)	−2	42°30.5940′	27°22.075′	2463	27.9	9.22	0.15	0.490	17	7.69	0.50	0.26
Lake Uzungeren (IBW0710)	−3	42°26.1551′	27°27.2235′	177	27.6	8.45	0.45	0.175	1132	9.7	0.40	0.28
Res. Zhrebchevo (IBW2545)	253	42°36.6024′	25°51.2345′	1851	27.6	7.70	0.7	0.358	233	8.01	0.10	0.18
Res. Duvanli (IBW1483)	250	42°23.1851′	24°43.1000′	27	26.3	8.76	0.4	4.050	291	7.09	0.10	0.25
Res. Sinyata Reka (IBW1890)	317	42°28.1480′	24°42.217′	6	27.4	9.72	0.5	0.470	305	9.36	25	4.8

## Data Availability

The data presented in this study are openly available in [NCBI] at [https://www.ncbi.nlm.nih.gov/ (assessed on 26 May 2021)], reference number [MZ274329-MZ274337].
